# Towards a Treatment for Intolerance of Uncertainty for Autistic Adults: A Single Case Experimental Design Study

**DOI:** 10.1007/s10803-018-3550-9

**Published:** 2018-03-27

**Authors:** J. Rodgers, R. Herrema, E. Honey, M. Freeston

**Affiliations:** 1 Clinical Psychology, Faculty of Medical Sciences, Institute of Neuroscience, Sir James Spence Institute, Newcastle University, Royal Victoria Infirmary, Queen Victoria Road, Newcastle, NE1 4LP UK; 2 School of Psychology, Faculty of Medical Sciences, Newcastle University, Newcastle, UK; 3 Northumberland, Tyne and Wear NHS Foundation Trust, Newcastle upon Tyne, UK

**Keywords:** Autism Spectrum Disorder, ASD, Adult, Anxiety, Intolerance of uncertainty, Intervention

## Abstract

Intolerance of uncertainty (IU) is indicated as an important transdiagnostic process variable in a range of anxiety disorders. Anxiety is very common in autistic adults. This study evaluates a manualised treatment programme for autistic adults, which focused on IU. An eight session programme (CUES-A©) was developed and delivered to four autistic adults on an individual basis. A single case experimental design was used to provide a preliminary evaluation of the feasibility, acceptability and preliminary effectiveness of the programme. Data regarding retention, acceptability and feasibility indicate that the participants valued the programme. Analyses of outcome measures indicate that the programme has promise as a treatment option for autistic adults experiencing IU.

**Electronic supplementary material:**

The online version of this article (10.1007/s10803-018-3550-9) contains supplementary material, which is available to authorized users.

## Introduction

Around 50% of autistic people experience levels of anxiety that affect their everyday lives, highlighting the need for effective treatments (Davis et al. 2011; Sterling et al. 2008; Mazefsky et al. 2008). Autistic individuals frequently present with multiple anxiety disorders concurrently, therefore treatments targeting underlying mechanisms may be most efficacious. A recent evidence-based theoretical framework to explain the mechanism that confers increased vulnerability to anxiety and to inform treatment in ASD has been proposed that includes intolerance of uncertainty as an important transdiagnostic mechanism (South and Rodgers 2017). The intolerance of uncertainty model of anxiety (Dugas et al. 1998) identifies IU as an assumption that uncertainty is stressful and upsetting and not knowing what is going to happen is negative and should be avoided at all costs. IU is considered to be a ‘broad dispositional risk factor for the development and maintenance of clinically significant anxiety’ (Carleton 2012). It involves the ‘tendency to react negatively on an emotional, cognitive, and behavioural level to uncertain situations and events’ (Buhr and Dugas 2009). Individuals who are intolerant of uncertainty find uncertain situations stressful and upsetting; have a tendency to interpret all ambiguous information as threatening and find it difficult to function in the face of uncertainty (Buhr and Dugas 2002, 2009; Laugesen et al. 2003). Indeed, uncertainty itself is perceived as threatening by people high in IU (Carleton 2012). IU has been linked to the development and maintenance of worry and Generalised Anxiety Disorder (GAD) (Buhr and Dugas 2006, 2009, 2012; Dugas et al. 1997, 2005; Freeston et al. 1994) and has also been proposed as a key underlying process in Obsessive Compulsive Disorder (OCD) (Holaway et al. 2006; Sookman and Pinard 2002; Tolin et al. 2003). More recently, IU has been linked to other disorders, including social anxiety disorder (Boelen and Reijntjes 2009; Carleton et al. 2010), panic disorder (Boswell et al. 2013) and anxiety sensitivity more generally (Carleton et al. 2007). IU is clearly important in the development and maintenance of anxiety in the general population.

Recently, research has begun to investigate the importance of IU to anxiety in Autism Spectrum Disorder (ASD). The concept resonates clinically with some of the core characteristics of ASD (Joyce et al. 2017; Rodgers et al. 2012; South and Rodgers 2017). Restricted and repetitive behaviours, such as insistence on sameness, inflexible adherence to routines and difficulty tolerating change have been linked with anxiety since the earliest descriptions of the disorder (Kanner 1943). These behaviours bear a conceptual resemblance to IU, with its associated avoidance of unexpected events and the desire to make life as predictable as possible (Rodgers et al. 2012). Evidence is now emerging that IU has a central role in the relationship between ASD and anxiety. Boulter et al. (2014) modelled the relationship between anxiety and IU in an ASD group and a neurotypical comparison group. Results confirmed significant relationships between IU and anxiety in autistic children and were consistent with a causal model, suggesting that IU mediates the relationship between ASD and anxiety. Wigham et al. (2015) examined the role that IU has in pathways between sensory processing difficulties, anxiety and restricted and repetitive behaviours (RRB) in ASD. These relationships were mediated by IU, indicating the important role IU may have in the interaction between anxiety and ASD traits. This is further supported by Neil et al. (2016) who reported that IU is an important construct to explain the relationship between sensory sensitivities and anxiety in autistic children. Chamberlain et al. (2013) report associations between shared neurobehavioral mechanisms in ASD and anxiety, indicating specific avenues for intervention targeting IU, and Maisel et al. (2016) illustrate the role that IU has in anxiety in autistic adults.

In terms of assessment, Rodgers et al. (2016b) developed and validated a child self and parent report measure of anxiety for autistic young people (the ASC-ASD). Using factor analytic techniques, the study identified four valid anxiety subscales, including an *uncertainty* scale. Hodgsonet al. (2017) undertook focus groups with parents of autistic young people exploring the concept of IU. Parents differentiated IU from dislike of change and from fear, discussed examples of IU and its impact on their children, and suggested that IU is a recognisable and important construct associated with anxiety that is distinguishable from but related to features of ASD. Kerns et al. (2016) in a discussion of the differential diagnosis of anxiety disorders in autism report that *fears associated with uncertainty* may be an important mechanism in the development and maintenance of anxiety in ASD. In conclusion, this evidence indicates that IU is an important mechanism in the development and maintenance of anxiety for autistic people and, as for neurotypical people (see Einstein 2014) an appropriate target for intervention.

The concept of IU has utility not only to theoretically inform understanding of factors underlying development and maintenance of anxiety, but has also been shown to be a beneficial target for treatment. Research has shown that experimental manipulation of intolerance of uncertainty can affect levels of worry in non-clinical neurotypical participants (Ladouceur et al. 2000). Cognitive behavioural treatments for clinically anxious patients have been developed which emphasise treating the cognitive *process* rather than the cognitive *content* of anxiety, specifically by aiming to increase patient’s tolerance for uncertainty and thereby achieving more sustainable change (Wilkinson et al. 2011). Research has confirmed the utility of such CBT protocols in reducing anxiety both in individual (Dugas and Ladouceur 2000; Ladouceur et al. 2000) and group formats (Dugas et al. 2003). Case series have also demonstrated the successful use of this intervention with neurotypical children and adolescents (Leger et al. 2003; Payne et al. 2011).

A variety of cognitive behaviour therapy (CBT) based programmes for anxiety and ASD have been evaluated recently, mainly in children and adolescents (Chalfant et al. 2007; McConachie et al. 2014; White et al. 2009; Wood et al. 2009) and with variable evidence for their effectiveness. These intervention programmes using CBT approaches have been variously adapted to meet the needs and learning styles of people with ASD. However, the application of these techniques, driven by the increasing awareness of the mental health needs of this population, is in advance of clear understanding of the underlying mechanisms inherent in anxiety in ASD. There remains much still to be done to specify models of anxiety for ASD populations, to enable the development of more targeted and effective intervention programmes. Importantly, Keefer et al. (2016) in a multisite manualised group intervention for autistic children with high anxiety in the USA demonstrated that high levels of pre-treatment IU significantly predicted poorer treatment response.

A parent based group intervention (CUES©: Coping with Uncertainty in Everyday Situations), aimed at providing parents of autistic children with effective strategies to reduce IU in their children in everyday situations has recently been developed and the intervention is reported to be acceptable and feasible to families (Rodgers et al. 2016a). However, there is a critical need to develop effective interventions specifically for autistic adults.

The aim of this study therefore was to adapt and provide a preliminary evaluation of the feasibility and acceptability of an adapted version of the CUES© intervention programme, aimed at reducing IU, to be delivered on an individual basis to autistic adults (CUES-A©).

## Method

### Participants

Autistic adults who had participated in focus groups (N = 12) from a previous research study investigating concerns about the future, were approached with information regarding this study and invited to take part. Four participants responded. Inclusion criteria was a clinical diagnosis of ASD, an adult (18+ years) and a self-reported difficulty in managing uncertainty. Exclusion criteria included the presence of a learning/intellectual disability or presence of a complex or severe mental health problem.

### Design

This research used a Single Case Experimental Design (SCED), which allows monitoring of change within participants and comparison between phases. Commensurate with SCED the study was conducted over three phases; baseline (A), intervention (B) and follow-up (C). Baseline length was a minimum of 5 days to ensure sufficient data points and to establish stability.

### Measures

#### Social Responsiveness Questionnaire-2A (SRS-2A)

Participants completed the SRS-2A during the initial meeting with the therapist. The SRS-2A (Constantino and Gruber 2012) is a standardised questionnaire used extensively to rate the social communication difficulties of autistic adults. The measure quantifies severity of autistic characteristics and can be used to monitor symptoms throughout the life span (Frazier et al. 2012). A Total Score in the range of 60–65 indicates an individual to be within “mild range” indicating deficiencies which are clinically significant and may lead to mild to moderate interference with everyday social interactions. A total score within the range of 66–75 indicates an individual to be within the “moderate range”, these scores are typical for individuals with an ASD of moderate severity. A total score of 76 or higher indicates an individual to be within the “severe range” leading to severe and enduring interference with everyday social interactions.

#### Primary Outcome Measure: Target Situation Monitoring

SCED approaches use repeated measures in each of the phases to allow comparison across phases, usually comprising of daily measurement using individualised diaries. In order to measure the degree and process of change, participants undertook individualised self-monitoring of personally relevant anxiety symptoms, target behaviours and engagement in target uncertain situations. Participants completed a very brief daily diary during all three phases: Baseline (Phase A—at least 5 days prior to commencement of intervention), intervention (Phase B—8 weeks) and follow-up (Phase C—at least 4 weeks after completion of the intervention). The diary included brief Likert scales delivered via a range of different methods, dependent on participant preference (e.g. email, online survey, spreadsheet or text message prompts), and took approximately 5 min to complete. Questions were individualised for each participant, who chose their own emotive anchor word (e.g. stressed, anxious, frustrated) and their own scale (e.g. 1–5, 1–10). Some scales were operationally defined for each individual by adding anchor labels to enable further understanding and effective use of the scale. An example of the template for the diaries is shown below.

#### Daily Diary Template

1. How anxious (emotion) do you feel about the target situation (0–100% or scale)?
2. If you were to experience your target situation, how anxious would this make you feel (0–100%)?
3. How confident do you feel about tackling your target situation (0–100%)?
4. How well do you think you could handle your target situation (0–100%)?
5. Has anxiety about your target situation stopped you from doing anything today? Yes/No
  - If Yes, what have you avoided doing?
6. Did your target situation occur? Yes/No
  - If Yes, how well did you handle the situation? (0–100%)
7. How anxious have you felt generally today? (0–100%)
8. Have you used techniques discussed in sessions today? Yes/No
  - If Yes, was this in relation to your target situation? Yes/No
9. Has feeling anxious/uncertain stopped you from doing anything today? Yes/No
  - If Yes, what did you avoid?

#### Secondary Outcome Measures

The following measures were taken on three occasions; at baseline, at the first session of the intervention and 4 weeks after the end of the intervention.

#### Patient Health Questionnaire 9 (PHQ-9)

The PHQ-9 (Martin et al. 2006) is a 9-item depression sub-scale from the Patient Health Questionnaire. It is used to assist clinicians with screening for depression and monitoring treatment response. The items of the PHQ-9 are based directly on the nine diagnostic criteria for major depressive disorder in the DSM-IV. The indicative clinical cut-off score utilised was 9.

#### Generalized Anxiety Disorder 7 (GAD-7)

The GAD-7 (Spitzer et al. 2006) is a 7-item self-reported *questionnaire* for screening and severity measuring of *generalized anxiety disorder*. The indicative clinical cut-off score utilised was 10.

The PHQ-9 and GAD-7 are the most widely used questionnaires in Primary Care Mental Health Services to assess levels of low mood and anxiety.

#### Intolerance of Uncertainty Scale (IUS-12)

IUS-12 is short 12-item scale questionnaire which screens for anxious and avoidant components of IU (Carleton et al. 2007). The 12 items are rated on a 5 point Likert scale ranging from 1 (not at all characteristic of me) to 5 (entirely characteristic of me) with a total score ranging from 12 to 60. Based on data reported by Carleton et al. (2012), a score of 35 in adults is the point where the non-clinical and clinical distributions intersect. The intersection conceptually represents criterion “c” as defined by Jacobson and Truax (1991). Therefore a score of > 35 may be a meaningful indicator of “Significant IU” in adult samples.

#### Stress Scale from Depression, Anxiety and Stress Scale (DASS-21)

The DASS-21 (Lovibond and Lovibond 1995) is a 21-item scale which screens for depression, anxiety and stress. There are seven items on each sub-category. Scores can range from 0 to 42 for each sub-category. A higher score indicates higher severity. The main use of the DASS is to assess the degree or severity of an individual’s depression, anxiety and stress. As the PHQ-9 and GAD-7 are already measuring depression and anxiety, the stress scale was exclusively used in order to solely measure stress levels for participants. Crawford and Henry (2003) report the reliability of the DASS-21 to be excellent. There is no clinical cut off for the stress subscale of the DASS.

#### The Adult Repetitive Behaviour Questionnaire-2 (RBQ2-A)

The RBQ2-A (Barrett et al. 2015) has been adapted from the Repetitive Behaviours Questionnaire-2, which was designed for parents of young children to use. The RBQ2-A is a 20-item questionnaire used in clinical practice to assess the frequency of repetitive behaviours in autistic adults. The items are rated on a 3 or 4 item scale. There is no clinical cut off for the RBQ2-A.

#### Feasibility and Acceptability Interviews (Conducted in Phase C)

This phase occurred at least 4 weeks after the participant’s final intervention session. Participants were asked to complete primary outcome measures daily over the course of 1 week prior to their follow-up session and secondary outcome measures on one occasion. Once participants completed the intervention phase and all follow up measurements, they were interviewed about their experiences of CUES-A©.

#### CUES-A© Procedure

All participants followed the same procedure, although may have progressed through sessions at a different rate. Participants attended an initial session, followed by a minimum of 5 days break (Phase A). Participants then commenced eight or nine therapy sessions, depending on individual need (Phase B). The sessions were designed to specifically address a target uncertain situation identified by the participant, which they found difficult to manage and caused some degree of negative affect. Following the therapy sessions, participants had at least a 4 week break followed by a final follow-up session (Phase C). All sessions were audio recorded for analysis.

#### Baseline—Phase A

Phase A included completion of baseline measures for each participant. Participants met the therapist initially to discuss individualisation of the primary outcome measure. Participants then began daily target monitoring (primary outcome measure). A minimum of 5 points per phase is considered the standard in multiple baseline design (Smith 2012). This is extended on an individual basis if a baseline pattern is unclear. Participant 1 had 6 days of baseline data points, Participant 2 had 11 days of baseline data points, Participant 3 had 12 baseline data points, and Participant 4 had 21 baseline data points.

#### Treatment-Phase B—Delivery of CUES-A© Programme

The treatment phase was the delivery of the manualised intervention (CUES-A©) facilitated by a member of the research team who is qualified in low intensity psychological therapies (PGCert) and has experience of working with autistic individuals. Based on the Coping with Uncertainty in Everyday Situations programme (CUES©), Rodgers et al. (2016a), eight to nine individual sessions were delivered to each participant, lasting approximately 1 h each. Participants completed the secondary outcome measures in their initial intervention session and completed primary outcome measures daily throughout this phase.

The content of the CUES-A© Programme includes: familiarisation with the intolerance of uncertainty (IU) model of anxiety, strategies for identifying anxious thoughts, understanding the consequences of IU, the relationship between IU and characteristics of autism, behavioural techniques to increase tolerance of uncertainty, generalisation and maintenance of strategies. Participants completed tasks outside the sessions as agreed collaboratively within sessions. The specific content of CUES-A© was adapted to the specific needs of each individual, based on their particular presentation and capabilities. The programme is designed to be flexible and incorporate personal information that participants chose to share. A bespoke range of strategies based on individual presentations were discussed with participants. These strategies are based on CBT techniques used in evidence-based interventions but adapted for IU. All participants received psycho-education on CBT and IU, as well as some combination of cognitive re-structuring, mindfulness, behavioural experiments and relapse prevention. The therapist received regular supervision from a Clinical Psychologist to ensure safe, effective and reflective practice. All sessions were audio recorded for analysis, alongside written notes taken during each session, including reflections. The therapist also completed a fidelity to delivery checklist after each session to ensure reliability of the delivery of each session.

The main components of the intervention are described below:

##### Initial Session

During the initial session the therapist met with the participants to discuss issues related to confidentiality, consent and risk. Psychoeducation relating to intolerance of uncertainty was provided and the participant and therapist together identified a target uncertain situation that would comprise the primary outcome measure, discussed an agreed the format for the daily diary and completed the secondary outcome measures.

Session 1: Topics covered during session one included a further introduction to CUES-A^©^ and Intolerance of Uncertainty (IU); goal setting; psychoeducation relating to formulation.

Session 2: Session 2 incorporated a review of the previous week and the use of the daily diary (also included in all subsequent sessions); consideration of the relationship between IU and autism; identification of barriers and less helpful strategies; discussion of intervention choice.

Sessions 3–7: These sessions incorporated intervention delivery based on the individuals presentation and could include any combination of cognitive re-structuring, behavioural experiments, mindfulness.

Session 8: This session focused on review, relapse prevention and consolidation.

#### Phase C—Follow-Up

**Fig. 1:**
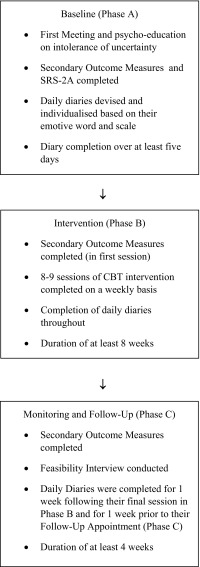
Flow chart of research procedure

Participants were followed up at least 4 weeks after their final session in Phase B. Participants were able to attend their follow-up session either over the phone or during a face-to-face appointment. Daily monitoring (primary outcome measures) continued for a week following their final appointment in Phase B and a week prior to their follow-up appointment with a 2 week break with no contact in between. secondary outcome measures were completed at the follow-up appointment. Participants were then invited to participate in a semi-structured interview about their experiences of the programme, allowing for further exploration of their positive and negative feedback regarding the programme and how effective they found it to be. Figure 1 outlines the research procedure.

#### Ethical Considerations

A favourable ethics opinion was provided by Newcastle University, UK, Faculty of Medical Sciences Ethics Committee. All participants provided informed, written consent. Participants collaboratively completed a Normal Operating Procedure template to ensure their safety regarding risk of harm to self or others and included information regarding local support services and emergency numbers. Risk was reviewed in every session and any changes in risk level were addressed and responded to accordingly. Risk was discussed during supervision to ensure participant safety and to formulate risk management plans where appropriate. Participants were also offered thorough assistance in referring to psychological services if required, to support their engagement and transition into available support services. This was offered on an individual needs basis for participants who had expressed additional difficulties, which were not possible to address within the structure and purpose of this intervention.

## Results

### Attendance, Retention and Completion

Retention to the intervention was 100%, all participants attended all scheduled sessions with short breaks for holidays, illness or travel, and completed all outcome measures.

### Case Study Descriptions

Further detailed information can be found about each participant and their tailored intervention below.

#### Participant 1

This participant has two autistic children and her target uncertain situation related to their children’s social interactions with others, which may lead to her having to interact with others unexpectedly. This covered a variety of settings, including school, hobbies, friend’s parents and strangers. Participant 1 expressed that she is often misunderstood or struggles in social situations and therefore these situations can have an uncertain and potentially negative outcome.

Target situation:* My children making me interact with someone I don’t know*.

Scale: Stress (0–10): Participant 1 chose to operationalise each number on the scale in order to make it concrete and meaningful to her.

Intervention treatment: A personalised strategy was developed to challenge thoughts and put situations into perspective. Participant 1 was encouraged to problem solve whether situations need an immediate response on all occasions or whether sometimes it is best to wait and re-evaluate. She described how sometimes she made impulsive decisions in order to create certainty.

A personalised alternative strategy was developed and captured by the acronym DIRECT.

D: Danger, is my child in danger? If the answer is yes, intervene to ensure their safety. If no, then continue with strategy.
I: Identify the issue, ask “what am I nervous about?” identifying what is happening internally and externally.
R: Responsibility to react—ask “Do I need to intervene in order to minimise distress to my children or others?” If yes, intervene, if no, continue to monitor.
E: Explain “Actions”—ask “What would I say if someone asked me why I’m not doing anything? How would I explain my decisions/actions to someone else?”
C: Conscious of my stress—bring awareness and focus of attention back on situation—back to what is happening internally and externally.
T: Tension—dealing with tension, calming down and focusing on breathing; ten mindful breaths.

#### Participant 2

This participant was in the process of setting up his own business enterprise which will be a support service for autistic adults; therefore this was a significant period of stress and anxiety for him. He was having difficulty coping with the elements of this process that were out of his direct control and his target situation was therefore related to the impact of other people’s decision making on him and inconsistent engagement.

Target situation: Other people making decisions that might impact on me and also my business enterprise.

Scale: Frustration (0–4.5).

1: Not frustrated at all
2: “Couldn’t give a ****”—indifferent to situation
3: Frustrated enough to take impulsive action
4: Too frustrated to do anything—avoidant
4.5: Meltdown—completely incapable of coping

Intervention treatment: Mindfulness, Relaxation strategies—progressive muscle relaxation, strategies to manage IU—particularly when not in control of the uncertain situation. These strategies were used in order to decrease initial arousal level and prevent impulsive responding, allowing him to “sit” with the uncertainty for some time. He used the following acronym to approach the situation:

Pause**—**Don’t act straight away.

Acknowledge and assess.

Contemplation—Why is this affecting me? Think about the situation as a whole.

Control—Can I do anything about it? Am I in control of the situation?

Change, accept or let go?

#### Participant 3

This participant had other difficulties relating to anxiety and depression, self-esteem and self-worth. He was therefore assisted in seeking some more general support from a psychological service following his involvement in the intervention.

Target situation: Sudden or unexpected changes to routine that cause anxiety, mainly but not exclusively relating to work.

Scale: Anxiety (1–10) This client did not describe his scale in as much detail as the other participants but explained that a score of “10” would be considered worst case scenario, when he would be experiencing sensory difficulties and would most likely isolate himself.

Intervention treatment: Cognitive re-structuring and mindfulness. Some examples from cognitive re-structuring are shown below.

First example: at home, uncertainty about the impact of being late for work, feeling anxious, negative thought: “I’m going to be late for work at 9 a.m.”—what might happen if I am late for work? People will think I’m not good at my job.

Evidence for: got up late.

Evidence against: Takes 20 min to get to work, alternative thought “plenty of time to get to work and core hours don’t start until 9.30 a.m.”

Second example: at home, uncertainty about whether an external agency will automatically deduct money from bank account, feeling anxious, negative thought: last year’s home contents insurer automatically renewed my policy and tried to take my money, will they pursue it? Evidence for: they sent me an e-mail.

Evidence against: Distance selling regulations, alternative thought: I’ll be able to cancel.

For this participant we encouraged lots of use of questioning, recognising that it is helpful to ask “is this going to impact negatively on me?” It was also recognised that it was useful to encourage him to also ask; what do I already know about this/what is my past experience of this? From looking at the facts, what is the alternative? Am I over-thinking it? Participant 3 found that by asking some of these questions, he was able to re-frame his thoughts more realistically in relation to uncertainty.

#### Participant 4

This participant had just begun university for the second time. He had previously attempted university 14 years previously aged 19 years. He had dropped out after 1 year and one semester due to barriers he described as presented by his autism diagnosis and lack of accessibility to appropriate support from the university to manage this. The timing of the CUES-A© programme came at a time of particularly elevated anxiety as he began his university career once again, and his social and environmental situation drastically changed during the research intervention. He related that the intervention had come at the best time in some ways as he was going through such a big transition and changes in his life, but that this also meant it was the worst time for the intervention as he had to cope with so much change during this time.

Target situation: Social interactions with others and feeling unsure about the authenticity of other people’s reactions.

Scale: Anxiety (1–5) Participant 4 identified that he would never score himself as a “0” or a “5”, he explained that a “0” would be when he is on holiday and has no worries at all for at least the week ahead. Whereas, “5” would be if he was completely unable to cope or do anything about the situation, a full “meltdown” leading to aggressive or damaging behaviours, he would notice tension, feeling agitated, noticing a feeling in his stomach, also having visual problems and sensitivity to noise and light, and this would make him withdraw.

Intervention treatment: Identifying causes and consequences of uncertain situations, evaluating rumination and when to stop this evaluating whether it is helpful and strategies to acceptance of uncertainty and mindfulness.

This participant created his own strategy:

1. Awareness: non-judgemental observation.
2. Label and Release: naming/acknowledging emotion.
3. Experience the moment/breathe.

### Visual Analysis

**Fig. 2:**
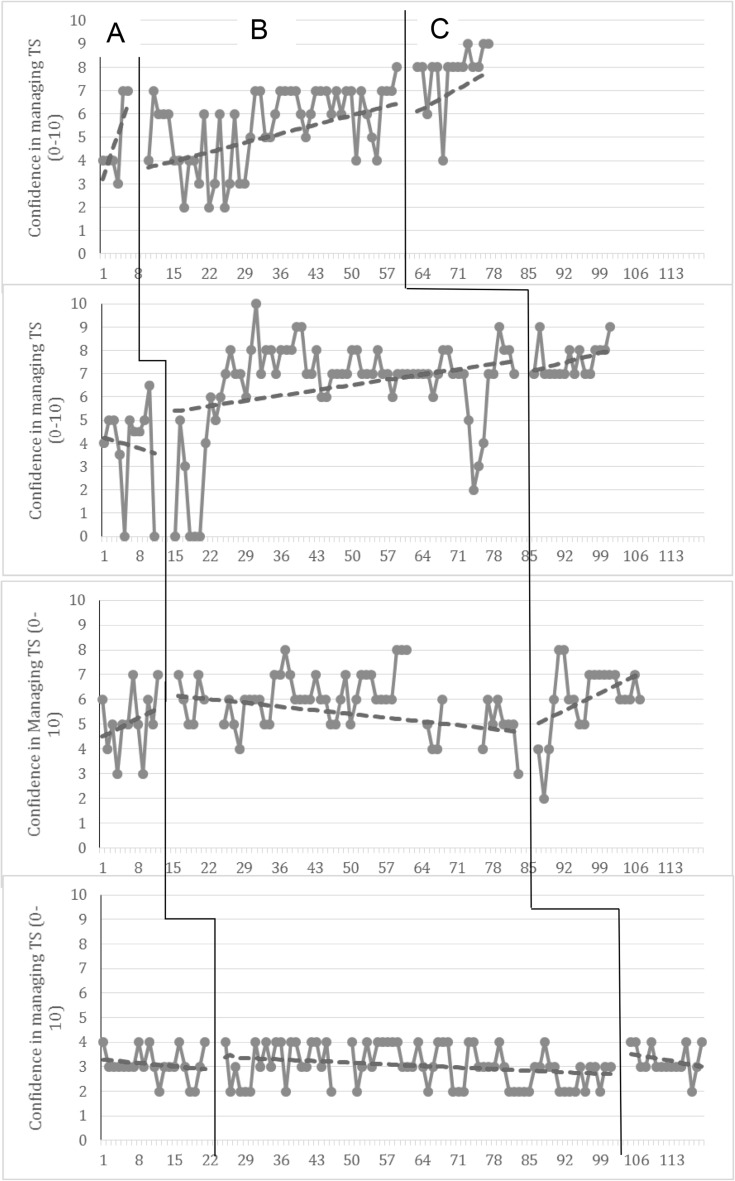
Daily diaries in relation to target situation. Question: How confident do you feel about tackling your target situation?

In line with SCED design, results are presented in graphical format based on questions from the daily diaries across all phases, ordered from shortest to longest baseline lengths; data from participants is presented in the same way in Table 1. Graphs have been adapted so that all x axes show the same information and all y axes show the scale participants used within their daily diary. The variable depicted in the graphs is *confidence in managing their target uncertain situation* (Fig. 2). Given the early stage of the research cycle and the aims of the study relating to feasibility and acceptability we selected one primary outcome to report here. *Confidence in managing the target uncertain situation* was selected as our primary outcome measure because the main goal of the programme is to promote the use of helpful strategies to be able to more successfully manage uncertain situations in the future. Given this aim assessing increased confidence in managing uncertainty, especially in frequently occurring situations in areas of occupational or family functioning, is essential to a determination of the usefulness of the intervention and indeed is likely a pre-requisite for the additional outcomes such as perceptions of success in managing uncertainty and changes in distress related to uncertain situations. Visual data from the graph were rated by two researchers (one independent of the study team) with 100% inter-rater reliability.

**Table 1:** Participant demographics

	Gender	Age	Age of diagnosis	Diagnosis given	Occupation	Ethnicity	SRS-2A Score
Participant 1	Female	39	34	ASD	Employed full-time	White-American	73 (moderate)
Participant 2	Male	30	27	High Functioning Asperger’s	Unemployed—works voluntarily	White-British	60 (mild)
Participant 3	Male	36	12	Asperger’s Syndrome	Employed full-time	White-British	84 (severe)
Participant 4	Male	33	30	Asperger’s Syndrome	Student	White-British	78 (severe)

### Statistical Evaluation of Change

Tau-U (Parker et al. 2011) is an estimate of non-overlap between phases; the greater the degree of non-overlap, the greater the difference between the scores in each phase. Tau-U can be corrected for baseline-trend. Tau-U is first calculated for each participant and then an aggregate value is calculated, weighted by the length of the series. Table 2 shows that two of the eight individual phase comparisons (A vs. B and B vs. C) were significant. When data from all participants were combined, significant increases in confidence ratings from Phase A to Phase B and from Phase B to Phase C can be seen (Table 2).

**Table 2:** Tau-U calculations across phases for each participant and all participants combined

Phase	Phase A versus B				Phase B versus C			
Participant	Tau	SD Tau	Z Score	p	Tau	SD Tau	Z Score	p
Participant 1	0.177	0.252	0.702	.482	0.272	0.171	1.588	.112
Participant 2	0.734	0.189	3.887	.001	0.069	0.161	0.433	.665
Participant 3	0.326	0.185	1.758	.078	0.273	0.149	1.829	.067
Participant 4	− 0.017	0.144	− 0.121	.904	0.590	0.164	3.589	.003
Combined	0.264	0.091	2.905	.004	0.298	0.080	3.706	.0002

### Standardized Measures

Table 3 shows the standardized outcome measures for each participant, at Initial Session and Follow-Up session. The “outcome” column indicates whether the Reliable Change Index has been met and so that the difference in their scores is “improved” as the scores have significantly decreased, or “recovered” as the scores have significantly decreased and moved from a clinical score to a sub-clinical score.

**Table 3:** Outcome measures for participants at initial session and follow-up

Scale	Participant 1			Participant 2			Participant 3			Participant 4		
	IS	FU	Outcome	IS	FU	Outcome	IS	FU	Outcome	IS	FU	Outcome
PHQ-9	4^a^	4	No change	10.5	4	Recovered	11	3	Recovered	16	12	No change
GAD-7	6^a^	0	Improved	9	7	No change	12	5	Recovered	18	14	No change
IUS-12	36	27	Recovered	43	32	Recovered	52	39	Improved	46	39	No change
Stress Scale	9	9	No change	15.5	7	Improved	18	3	Improved	15	11	Improved
RBQ-2A	38	26	Improved	56	44	Improved	38	26	Improved	49	42	Improved

^a^Indicates below clinical cut-off before treatment

### Feasibility and Acceptability Interviews

Data obtained from the Feasibility Interviews highlight a number of themes derived using Braun and Clarke’s (2006) method of thematic analysis.

- The flexibility of CUES-A programme was helpful.
- The individualisation of the programme was helpful.
- The collaborative approach was important.
- The language used was important.
- It would be helpful to have this support as well as additional practical support in relation to employment, sensory difficulties etc.

Some direct quotations from *participants during the feedback* interview regarding the CUES-A© intervention programme can be found below.

#### Participant 1

When we first started using the STOPP technique, they don’t go into whether you’re in danger and it didn’t address this, so it didn’t take into account your individual needs, which is why it was so good when we individualised our own.

I think I am using the strategies sub-consciously but generally feel a lot better when I’m having to deal with uncertainty.

#### Participant 2

I’ve come to realise I like uncertainty because it creates opportunities.

I felt I was able to contribute to sessions but it didn’t feel over-stated which is nice…it was good to have an honest and rational approach to sessions.

It’s been very supportive, and very hands on when it needs to be and very hands off when it needed to be. It was a very relaxing experience and I felt very comfortable and not judged at all.

#### Participant 3

I felt able to contribute to the sessions and I felt I could discuss any difficulties and reflect on my week and I felt able to say where the programme was going.

Learning techniques to manage uncertainty, particularly mindfulness and thought challenging were helpful.

#### Participant 4

The biggest tool which wasn’t actually formalised and was really helpful was the flexibility.

I don’t think I would have made the same progress if this had been done in a group.

I would recommend the programme to someone else if everything was changed in the sense that it was completely individualised and flexible to them.

This project (CUES-A) has enabled me to cope with things I wouldn’t have been able to previously.

There’s not so much of a flexible culture within IAPT/NHS services as there can be within a research programme. And those (mental health professionals) with maybe not as much support or less experience of people with autism, proper autism training with mental healthcare professionals would be hugely invaluable.

## Discussion

The aim of this study was to adapt and provide preliminary evaluation of the feasibility and acceptability of CUES-A©, delivered on an individual basis to autistic adults. Given the growing evidence base of the centrality of IU to anxiety in autistic people (Boulter et al. 2014; Chamberlain et al. 2013; Rodgers et al. 2016a, b; Keefer et al. 2016; Wigham et al. 2015), coupled with the high prevalence of multiple anxiety disorders occurring concurrently in ASD, targeting important transdiagnostic mechanisms, such as IU, may have significant treatment utility. The study builds on previous work developing a parent mediated intervention for autistic children with high IU (Rodgers et al. 2016a). Using a collaborative approach with four autistic adults, we co-constructed and then delivered a manualised, eight session intervention (CUES-A©). Utilising a single case experimental design we collected individual data monitoring change within participants and comparison between phases in relation to an individualised target uncertain situation, alongside a number of standardised measures. We also assessed feasibility and acceptability of the programme by recording attendance and completion, and through an end of programme evaluation questionnaire and interview. Attendance and retention to the programme was excellent. None of the participants dropped out and all participants indicated that they would recommend CUES-A to other autistic adults. Participants provided a range of free text comments in relation to the programme and a sample of these are provided here (the full data set is available on request from the corresponding author). In sum, these data indicate that the participants valued the programme, recognised the role of IU in their lives and found the strategies helpful.

Of course, given the stage of this work in the research cycle, the primary focus was on feasibility and acceptability. More formal evaluation of efficacy is a task for future studies.

The data from the single case experimental design are worthy of consideration here. All participants were able to generate and operationalise a target uncertain situation that they wished to address. Interestingly, for three of the participants the target situation related directly to difficulties which may be associated with an autism diagnosis, including difficulties with social communication, theory of mind and sudden changes to routine/plans, perhaps highlighting the increased vulnerability to IU that may be present for autistic individuals as a consequence of the interaction between autistic traits and IU (Wigham et al. 2015). Our primary outcome variable was the participant’s confidence in tackling their target uncertain situation. As can be seen from Fig. 2, for three of the four participants confidence generally increased over the course of the programme; this was maintained at follow-up, despite increased exposure to uncertainty as a consequence of engagement in behavioural experiments in relation to uncertainty that are a feature of participation in the programme. For Participant 4 the picture is not so clear cut. Figure 2 does not highlight a clear confidence increase in tackling the target IU situation for this individual; however, the CUES-A programme came at a time of particularly elevated anxiety for this participant, as he re-enrolled at university with all of the social and environmental changes that that confers. He related that the intervention had come at the best time in some ways as he was going through such a big transition and change in his life. As such, whilst we were not able to detect significant changes in confidence in relation to the IU target situation, Participant 4 reports that the programme enabled him to cope with things he would not previously have been able to manage. These conclusions are further supported by the Tau-U Calculations reported in Table 2. Tau-U was first calculated for each participant and then an aggregate value was calculated, weighted by the length of the series. Although only two of the eight individual phase comparisons were significant (Participants 2 and 4) perhaps due to low power, when all data were combined, a significant increase in confidence ratings from Phase A to Phase B and from Phase B to Phase C was found.

We also collected data at three time points using a range of standardised measures. Of course, with a sample of four participants, group based quantitative analyses of these data would not be meaningful. Rather we calculated reliable and clinically significant change for each participant. Two participants presented with scores on the PHQ-9, which assesses depressive symptoms, indicating recovery at follow-up (Participants 2 and 3). Participant 1 had low scores at baseline on this measure (below clinical cut-off and remained stable). Changes in self-reported anxiety and IU scores are, of course, of particular interest given the target of the CUES-A programme. Two participants showed change on the anxiety measure the GAD-7. Participant 1 (who was below clinical cut-off at baseline) showed improvement at follow-up, and Participant 3 showed reliable and clinically significant change and would be considered recovered at follow-up. Three participants demonstrated change in relation to the intolerance of uncertainty self-report measure (IUS-12). Participants 1 and 2 were considered recovered, whilst Participant 3, although still above clinical cut-off was improved. Similarly, three participants demonstrated significant improvement on the stress scale of the DASS-21. There is an emerging literature and theoretical framework suggesting a putative relationship between IU and restricted and repetitive patterns of behaviour for autistic individuals, such that engagement in repetitive acts may represent an attempt to impose certainty in an uncertain world (See South and Rodgers 2017). Given this emergent work, we were interested to determine whether participation in CUES-A may impact on self-reported engagement in RRB amongst autistic adults. To this end participants completed the RBQ2A. Whilst there is no clinical cut-off for the RBQ2A, we can see from Table 3 that all four participant’s scores on the measure indicate reliable improvement (i.e. less engagement) in repetitive behaviours at follow-up. To our knowledge this is the first time that a reduction in core autism symptomatology has been reported in relation to mental health intervention.

The lack of longer term follow-up is of course of note and whilst beyond the scope and goals of the current project is a major limitation of the current study. Of course, what is needed to determine the effectiveness of CUES-A© is a fully powered trial with long-term follow up of both proximal as well as more distal outcomes (e.g. quality of life, social or family functioning), in order to determine the clinical impact of the programme. With this in mind it will be important for future trials to think very carefully about the nature and timing of outcome assessments, and to develop a valid behavioural measure of IU to reduce the reliance on questionnaire measures of IU.

In summary, the current study sought to take the first steps towards the development of an intervention programme for autistic adults, which focuses on an important transdiagnostic construct underlying anxiety, intolerance of uncertainty. This preliminary evaluation of the acceptability and feasibility of the novel CUES-A© programme indicates that the programme is feasible to deliver directly to autistic adults, and is acceptable and face valid, and our preliminary data indicate that CUES-A has promise as a method to enable autistic adults to tackle the everyday challenges conferred by high levels of intolerance of uncertainty.

## Electronic supplementary material

Below is the link to the electronic supplementary material.

Supplementary material 1 (DOCX 12 KB)
