# Supplementary material for: Towards a Treatment for Intolerance of Uncertainty for Autistic Adults: A Single Case Experimental Design Study
Source: J Autism Dev Disord. 2018 Mar 27;48(8):2832–45. doi: 10.1007/s10803-018-3550-9 (PMC6061029; doi:10.1007/s10803-018-3550-9)
Supplement: Supplementary file 1 — Supplementary material 1 (DOCX 12 KB) [file 10803_2018_3550_MOESM1_ESM.docx]

**Appendix A. Supplementary data.**

Scale agreed for Participant 1

**0 –** No stress despite experiencing TS

**1** – Annoyed, but forgotten in seconds. (A mosquito buzzing near me)

**2**- Recovering in less than half an hour.

**3-** Feeling seriously unhappy, but realising that it could have been much worse, and thus making peace with my feeling of disease.

**4 –** A few hour of annoyance before naturally moving on.

**5 –** A few hours of rumination plus a sense that I need to process my feelings somehow, but I'm not quite crying. Might call my husband to vent etc.

**6 -** Crying

**7 –** Urgent need for escape at all cost. (Just getting up and walking away)

**8 –** Feeling like the rest of my day is ruined

**9**- Making a scene / feeling embarrassed

**10** – Doing or saying something that has long term negative effect (this has to be worse than feeling embarrassed)

**10+** - Acts of physical aggression
